# The effect of inhibition on rate code efficiency indicators

**DOI:** 10.1371/journal.pcbi.1007545

**Published:** 2019-12-02

**Authors:** Tomas Barta, Lubomir Kostal

**Affiliations:** 1 Institute of Physiology of the Czech Academy of Sciences, Prague, Czech Republic; 2 Charles University, First Medical Faculty, Prague, Czech Republic; 3 Institute of Ecology and Environmental Sciences, INRA, Versailles, France; University of Pittsburgh, UNITED STATES

## Abstract

In this paper we investigate the rate coding capabilities of neurons whose input signal are alterations of the base state of balanced inhibitory and excitatory synaptic currents. We consider different regimes of excitation-inhibition relationship and an established conductance-based leaky integrator model with adaptive threshold and parameter sets recreating biologically relevant spiking regimes. We find that given mean post-synaptic firing rate, counter-intuitively, increased ratio of inhibition to excitation generally leads to higher signal to noise ratio (SNR). On the other hand, the inhibitory input significantly reduces the dynamic coding range of the neuron. We quantify the joint effect of SNR and dynamic coding range by computing the metabolic efficiency—the maximal amount of information per one ATP molecule expended (in bits/ATP). Moreover, by calculating the metabolic efficiency we are able to predict the shapes of the post-synaptic firing rate histograms that may be tested on experimental data. Likewise, optimal stimulus input distributions are predicted, however, we show that the optimum can essentially be reached with a broad range of input distributions. Finally, we examine which parameters of the used neuronal model are the most important for the metabolically efficient information transfer.

## Author summary

Neurons communicate by firing action potentials, which can be considered as all-or-none events. The classical rate coding hypothesis states that neurons communicate the information about stimulus intensity by altering their firing frequency. Cortical neurons typically receive a signal from many different neurons, which, depending on the synapse type, either depolarize (excitatory input) or hyperpolarize (inhibitory input) the neural membrane. We use a neural model with excitatory and inhibitory synaptic conductances to reproduce in-vivo like activity and investigate how the intensity of presynaptic inhibitory activity affects the neuron’s ability to transmit information through rate code. We reach a counter-intuitive result that increase in inhibition improves the signal-to-noise ratio of the neural response, despite introducing additional noise to the input signal. On the other hand, inhibition also limits the neuronal output range. However, in the end, the actual amount of information transmitted (in bits per energy expended) is remarkably robust to the inhibition level present in the system. Our approach also yields predictions in the form of post-synaptic firing rate histograms, which can be compared with in-vivo recordings.

## Introduction

Cortical neurons receive input in the form of bombardment by action potentials (spikes) from other neurons and process and communicate the received information further by transmitting their own action potentials to other neurons. Individual action potentials do not differ in their time course and therefore, from the information processing point of view, they can be seen as all-or-none events. The response to a particular stimulus is therefore represented by a spike train—a sequence of times when an action potential was produced [1].

According to the efficient-coding hypothesis [2], neurons are adapted to process the information from their natural surrounding efficiently. This inspired a number of studies based on optimality arguments (e.g., [3–9]), with the information efficiency usually being interpreted by the means of Shannon’s information theory [10].

Given that the cortex has only a limited energy budget and information transfer is costly [11–13], we expect that neurons balance information rates and energetic expenses. The idea of energy efficient neural coding was popularized by Levy and Baxter [14]. In their work they focus on the representational capacity of a noiseless population of neurons and show that optimizing the representational capacity *per spike* leads to low firing rates, typically observed in vivo. The introduction of realistic noise [15] and further biophysical details limits the analytical tractability and studies of noisy neurons are generally limited to numerical analyses of single cells and simplified populations.

Typical approaches to information-theoretical analyses of single cells are either the use of the direct method [16, 17] to evaluate the reproducibility of a response to a given stimulus or the computation of the mutual information between the stimulus and the response [18] and eventually evaluating the information capacity of the neuron as an information channel [19–21]. The attractiveness of information capacity stems from Shannon’s channel coding theorem which guarantees the existence of a code that is asymptotically able to transmit information at the rate given by the capacity [22]. See [23–26] for reviews of the use of information theory in computational neurosciences.

Both the direct method and capacity analysis can be extended to account for the metabolic expenses. One of the earliest efforts to relate the information capacity to the metabolic expenses is that of Laughlin et at. [27], where the Gaussian distribution of response variability is assumed for a cell encoding the stimulus in the graded potential. Balasubramanian [28] discussed the possibilities of applying the formalism of capacity of constrained channels [29] to neural systems and Polavieja [30, 31] showed that rate coding neurons [32] with additive response noise that the predicted shapes of post-synaptic firing rate (PSFR) distributions obtained from such formalism qualitatively match the experimentally measured distributions [33]. These inspiring results provided ground for investigating the information-energy balance for more realistic neuronal models, such as the Hodgkin-Huxley model [34] or a formal model based on an empirical stimulus-response relationship [35]. Studies concerning the efficiency of neurons employing different methods of information encoding have also been conducted (e.g., Leaky integrate and Fire with descending threshold [36], generalized inverse Gaussian neuron model [37–39]).

**Fig 1:**
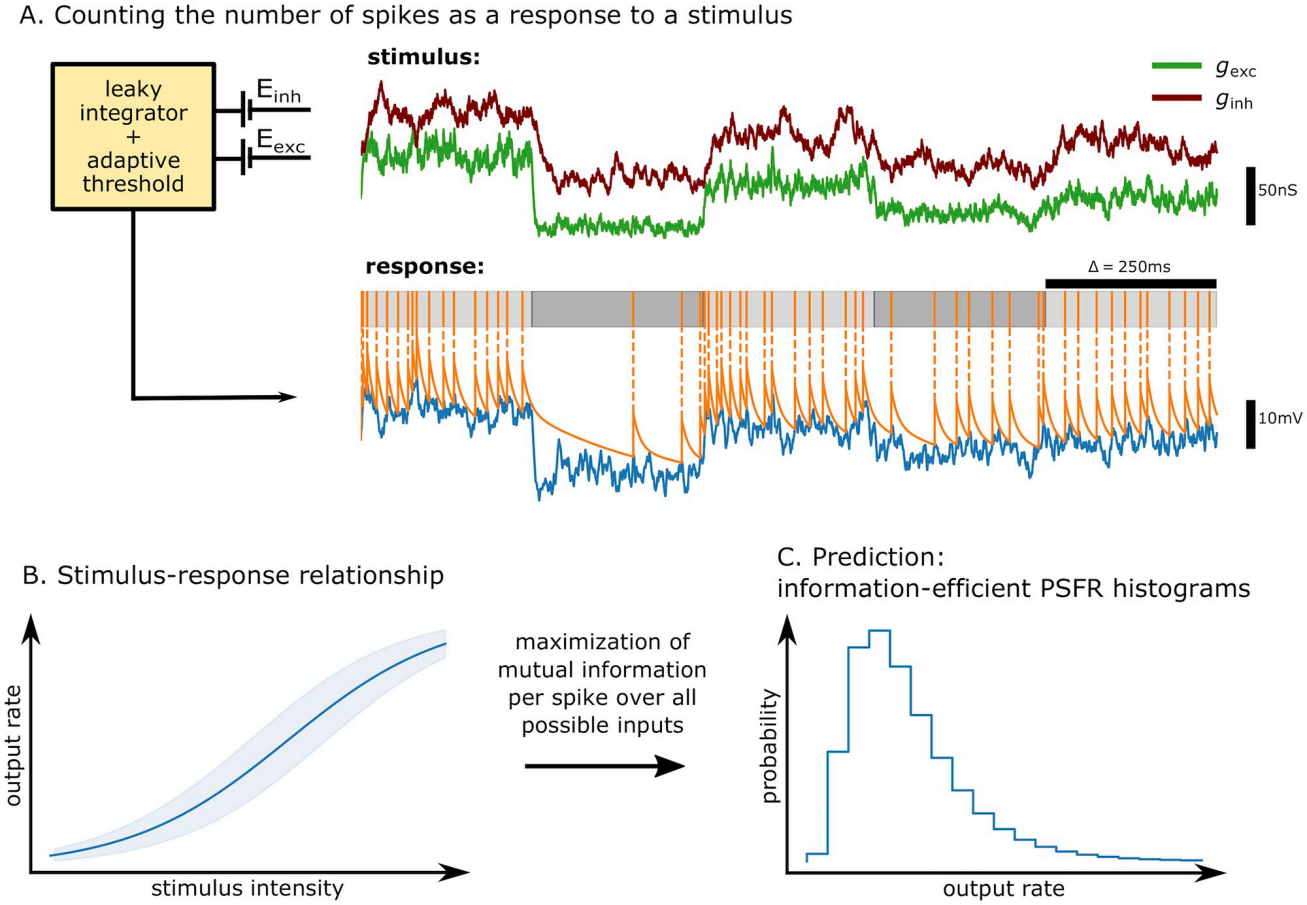
Graphical abstract. (A) Stimulus consisting of excitatory and inhibitory synaptic conductances, generated as shot noise with an exponential envelope, is delivered to the neuronal model, a passive leaky membrane with a dynamic threshold. The measured response is the number of spikes in a specified time window (e.g., 250 ms). (B) For each stimulus intensity the full response distribution is obtained. The mean response (solid) and its standard deviation (shaded) are shown for illustration. (C) We find the probability distribution of inputs that maximizes the mutual information between the stimulus and the response per single spike. The predicted histogram of post-synaptic firing rates (PSFR) can be compared with experimental data.

In the presented work we utilize the MAT (Multi-timescale Adaptive Threshold) model [40] which has been shown to be very good at predicting in-vivo recorded spike trains [40–47], while maintaining only a modest number of free parameters. Therefore information-theoretical analysis of this model allows us to make predictions for a wide variety of neurons (Fig 1).

The main contributions and the structure of this work can be summarized as follows:

1. By applying the results of Witsenhausen [48] in the context of neural systems, we conclude that the maximal mutual information between input and output of a neuron using rate code must be generally reachable with only a finite number of inputs.
2. We qualitatively discuss the stimulus-response relationships and the capacity-cost functions and show the stabilizing effect of inhibition on the membrane potential fluctuations and discuss the implications for the given neuronal model.
3. We analyze the effect of inhibition on information-metabolic efficiency and more intuitive indicators of information transmission efficiency. We find that for a given mean post-synaptic firing rate, counter-intuitively, increased ratio of inhibition to excitation generally leads to higher signal to noise ratio (SNR). On the other hand, the inhibitory input significantly reduces the dynamic coding range of the neuron.
4. We present the predicted PSFR histograms and discuss the comparability with experimental data. In combination with the relative simplicity of fitting the parameters of the MAT model to real neurons, the presented framework allows us to predict the PSFR histograms for a wide variety of neurons. Furthermore, we observe that the shapes of the histograms depend only marginally the rate coding time scale.
5. We show the predicted optimal input distributions and point out to the robustness of metabolic efficiency and the PSFR histogram towards changes in the input distribution.
6. We explain the effect of model parameters on the obtained results and the significance of the spontaneous firing rate. We use parameter values fitted by Kobayashi et. al. [40] on experimental data for further biological relevance and to provide insight into what influences the information-metabolic efficiency on a large scale.

## Materials and methods

### Neuronal model

The membrane potential of the MAT model is governed by the equation:

$$\begin{array}{c} \tau_{m}\frac{dV}{dt}=-\left(V-E_{L}\right)+RI_{\text{syn}}, \end{array}$$ (1)

where *τ*_m_ is the membrane time constant, *V* is the membrane potential, *E*_L_ = −80 mV the leakage potential, *I*_syn_ is the synaptic current. Spikes are fired when the membrane potential reaches (or is above) the value of a dynamic threshold *θ*(*t*). The dynamics of *θ* is described by

$$\theta (t)=\sum_{k}D\left(t-t_{k}\right)+\omega ,$$ (2)

$$D(t)=\sum_{j=1}^{L}H\left(t\right)\alpha_{j}\exp\left(-t/\tau_{j}\right)$$ (3)

where *k* iterates through all the previous spikes, *t*_*k*_ is the *k*-th spike’s time and *H* is the Heaviside function. Therefore the threshold is composed of *L* exponentially decaying components and an asymptotic threshold value *ω*. The *j*-th component increases by *α*_*j*_ every time a spike occurs and then decays with the time constant *τ*_*j*_. Absolute refractory period of 2 ms is introduced, during which the dynamics of the membrane potential and the threshold remain unchanged, but a spike cannot be fired. The parameters used to replicate the behavior of neurons from different classes (regular spiking—RS, intrisic bursting—IB, fast spiking—FS, chattering—CH) were identified by Kobayashi et al. [40]. All relevant model parameters are specified in S1 Appendix.

The synaptic current is given by

$$\begin{array}{c} I_{\text{syn}}\left(t\right)=g_{\text{exc}}\left(t\right)\left(V-E_{\text{exc}}\right)+g_{\text{inh}}\left(t\right)\left(V-E_{\text{inh}}\right), \end{array}$$ (4)

where *g*_exc_, *g*_inh_ are the total conductances of the excitatory and inhibitory synapses and *E*_exc_ = 0 mv, *E*_inh_ = −75 mv are the respective synaptic reversal potentials. We consider the excitatory and inhibitory conductances to be

$$g_{\text{exc}}\left(t\right)=\sum_{t_{j}<t}{\bar{g}}_{\text{exc}}H\left(t_{j}-t\right)\;\text{exp}\;((t_{j}-t)/\tau_{\text{exc}}),$$ (5)

$$g_{\text{inh}}\left(t\right)=\sum_{t_{k}<t}{\bar{g}}_{\text{inh}}H\left(t_{k}-t\right)\;\text{exp}\;((t_{k}-t)/\tau_{\text{inh}}),$$ (6)

where the times {*t*_*j*_}, {*t*_*k*_} are generated by independent Poisson point processes with intensities λ_exc_, λ_inh_ (to mimic the arrival of excitatory and inhibitory synapses), ${\bar{g}}_{\text{exc}}$ and ${\bar{g}}_{\text{inh}}$ correspond to peak conducatances of individual synapses and *τ*_exc_, *τ*_inh_ are time constants of those synapses, which were chosen as 3 ms for the excitatory and 10 ms for the inhibitory synapses [49]. We denote the excitatory part λ_exc_ as the *stimulus intensity* [34].

To recreate biologically plausible conditions, we calculate the peak conductances and minimal intensities of Poisson processes $\lambda_{\text{exc}}^{\left(\text{bcg}\right)}$, $\lambda_{\text{inh}}^{\left(\text{bcg}\right)}$ (where “bcg” stands for the background network activity), so that the mean and standard deviation of *g*_exc_ and *g*_inh_ correspond to values reported in [49], which were obtained from a detailed biophysical simulation. The values of the peak conductances are ${\bar{g}}_{\text{exc}}=1.50$ nS and ${\bar{g}}_{\text{inh}}=1.53$ nS and the rates of arrival of action potentials corresponding to the background activity are $\lambda_{\text{exc}}^{\left(\text{bcg}\right)}=2.67$ kHz, $\lambda_{\text{inh}}^{\left(\text{bcg}\right)}=3.73$ kHz (S3 Appendix).

The *response* of the neuron *y* is the number of observed spikes in a time window Δ, the corresponding firing rate is then *y*/Δ. Since the differential equation describing the membrane potential (Eq (1)) is stochastic due to the randomness introduced by the input current, the response is described by a random variable *Y* for each input λ_exc_. In our work we compare the results for five different lengths of coding time windows: 100 ms, 200 ms, 300 ms, 400 ms and 500 ms.

The numerical integration procedure is described in S2 Appendix.

### Metabolic cost of neuronal activity

The metabolic cost of neuronal activity is determined mainly by the activity of the Na^+^/K^+^ ionic pump in the neuronal membrane, pumping the excess Na^+^ out of the neuron. The main contributors to the overall cost are then: 1. reversal of Na^+^ entry at resting potential, 2. reversal of ion fluxes through post-synaptic receptors, 3. reversal of Na^+^ entry for action potentials and 4. additional costs associated with the action potential [12, 50, 51].

We follow the estimates from [11], i.e., we set the cost of maintaining the resting potential at *w*_rest_ = 0.342 ⋅ 10^9^ ATP molecules per second, the cost of reversal of Na^+^ entry for action potentials at 0.384 ⋅ 10^9^ ATP molecules per single action potential and the costs associated with vesicle release due to action potential at 0.328 ⋅ 10^9^ ATP molecules, adding up to *w*_spike_ ≈ 0.71 ⋅ 10^9^ ATPs/spike.

To calculate the cost needed to reverse the ion fluxes through post-synaptic receptors, we follow the approach used in [13]. We calculated the conductance of Na^+^ channels:

$$\begin{array}{c} g_{\text{Na}}=\frac{g_{\text{exc}}}{1-\frac{E_{\text{Na}}}{E_{K}}}, \end{array}$$ (7)

where *E*_Na_ = 90 mV, *E*_K_ = −105 mV are the reversal potentials of Na^+^ and K^+^ channels. The current due to influx of Na^+^ ions is then

$$\begin{array}{c} I_{\text{Na}}\left(t\right)=g_{\text{Na}}\left(t\right)\left(V\left(t\right)-E_{\text{Na}}\right). \end{array}$$ (8)

Integrating the current over Δ and dividing by the charge of an electron *e* gives us the total number of Na^+^ that have to be extruded. The ion pump uses one ATP molecule for 3 Na^+^ extruded.

Substituting *g*_Na_(*t*) and *V*(*t*) by their mean values (${\bar{g}}_{\text{Na}}\left(t\right)$, $\bar{V}\left(t\right)$) for excitation and inhibition intensities λ_exc_, λ_inh_, we obtain the approximate formula for the cost of reversal of the synaptic currents:

$$\begin{array}{c} w_{\text{syn}}\left(\lambda_{\text{exc}},\lambda_{\text{inh}}\right)=\frac{1}{3e}{\bar{g}}_{\text{Na}}\left(\lambda_{\text{exc}},\lambda_{\text{inh}}\right)\left(\bar{V}\left(\lambda_{\text{exc}},\lambda_{\text{inh}}\right)-E_{\text{Na}}\right)\Delta . \end{array}$$ (9)

The total cost of the signaling, given the input (λ_exc_, λ_inh_), is then:

$$\begin{array}{c} w\left(\lambda_{\text{exc}},\lambda_{\text{inh}}\right)=\left(w_{\text{rest}}+w_{\text{syn}}\right)\Delta +w_{\text{spike}}n\left(\lambda_{\text{exc}},\lambda_{\text{inh}}\right), \end{array}$$ (10)

where *n*(λ_exc_, λ_inh_) is the average number of spikes observed for the given input.

### Information capacity and capacity-cost function

In the framework of information theory, the input is a random variable *X* with probability density function *p*(*x*). In our case, *x* is the stimulus intensity, λ_exc_, which is a real number from an interval [*a*, *b*]. We can than define the corresponding marginal output probability distribution *q*_*p*_:

$$\begin{array}{c} q_{p}\left(y\right)=\int_{a}^{b}p\left(x\right)f\left(y|x\right)\;dx. \end{array}$$ (11)

The conditional probability distribution *f*(*y*|*x*) describing the probability of observing an output *y* given an input (stimulus) *x* has to be obtained first [22]. Due to the non-linear character of Eqs (1–6) the closed-form solution for *f*(*y*|*x*) is not available, therefore we used extensive Monte Carlo simulation to obtain the numerical approximation. The amount of information about the stimulus *X* = *x* from observing the response *Y* = *y* is defined as [22, p. 16]

$$\begin{array}{c} i\left(x;y\right)={\text{log}}_{2}\frac{f(y|x)}{q_{p}\left(y\right)}. \end{array}$$ (12)

By averaging the value of information over all possible outputs, we get the specific information (since *Y* is discrete) [52–54]

$$\begin{array}{c} i\left(x;Y\right)=\sum_{y=0}^{+\infty }i\left(x;y\right)q_{p}\left(y\right). \end{array}$$ (13)

By averaging the specific information over all possible inputs, we get the mutual information

$$\begin{array}{c} I\left(X;Y\right)=\int_{a}^{b}i\left(x;Y\right)p\left(x\right)\;dx. \end{array}$$ (14)

The *information capacity* expresses the maximal amount of information that can be reliably transmitted per single channel use and is defined as

$$\begin{array}{c} C=\underset{p(x)}{\sup}I\left(X;Y\right), \end{array}$$ (15)

where the supremum is taken over all possible input probability distributions. Since the duration of one channel use is Δ, $\frac{C}{\Delta }$ is the capacity in bits per second.

Given the input probability distribution *p*(*x*) the average metabolic cost *W*_*p*_ is then

$$\begin{array}{c} W_{p}=\int_{a}^{b}p\left(x\right)w\left(x\right)\;dx, \end{array}$$ (16)

where *w*(*x*) is given by Eq (10) We maximize mutual information over all possible input probability distributions *p* that satisfy the condition *W*_*p*_ < *W* for some selected *W*, and thus obtain the *capacity-cost function* [29]:

$$\begin{array}{c} C\left(W\right)=\underset{p\left(x\right):W_{p}<W}{\sup}I\left(X;Y\right). \end{array}$$ (17)

It follows from the Lagrangian theorem [55, 56], that *C*(*W*) is attained either at the cost corresponding to the unrestrained capacity *W*_max_ for *W* > *W*_max_ or at *W*. The quantity $\frac{C(W)}{W}$ for *W* ≤ *W*_max_ then expresses the amount of information per unit cost, which motivates the definition of *information-metabolic efficiency E* [28, 35, 57], i.e. the maximal amount of information per unit cost

$$E=\frac{C(W^{*})}{W^{*}},$$ (18)

$$W^{*}=\underset{W\in [0,+\infty )}{arg max}\frac{C(W)}{W}.$$ (19)

where W* is the optimal average cost.

We will refer to a regime in which the neuron encodes the maximal possible amount of information per energy as to an *information-metabolically efficient regime*. In such regime, the inputs *x* are assigned probabilities p*(*x*) and the probability of observing an output *Y* = *y* is

$$\begin{array}{c} P\left(Y=y\right)=\int_{a}^{b}p^{*}\left(x\right)f\left(y|x\right). \end{array}$$ (20)

Since the response *y* is the number of spikes in a time window Δ, we can use Eq (20) to calculte the mean PSFR:

$$\text{PSFR}=\frac{1}{\Delta }y$$ (21)

$$\left\langle \text{PSFR}\right\rangle =\frac{1}{\Delta }\sum_{y=0}^{+\infty }yP\left(Y=y\right).$$ (22)

### Properties of information-theoretic optima and numerical optimization

Theoretical results show that the support of the optimal input distribution p*(*x*) for certain channels (neuron with gamma distributed inter-spike interval [21], energy constrained Gaussian channel [56], Rayleigh-fading channel [58]) contains only a finite number of points. Moreover, as a consequence of Dubin’s theorem [48], it is guaranteed that for any channel with a finite number of possible output states the optimal input distribution has to be discrete. The number of support points is at most equal to the number of possible outputs. Since the number of action potentials in a finite time window is limited, it generally follows that the optimal input distribution in the rate-coding scheme must contain only finitely many stimulus values of non-zero probability.

The theory presented above holds for memoryless information channels without feedback, i.e., the response to the stimulus depends only on the current stimulus and not on any past stimuli or responses of the channel. However, real neurons exhibit adaptation to the stimulus, therefore the stimulus-response relationship *f*(*y*|*x*) is also affected by the probability distribution of stimuli *p*(*x*). In order to mitigate the effect of history, we developed a fixed-point based method to ensure that the distribution of stimuli *p*(*x*) used to obtain *f*(*y*|*x*) is the same as the predicted optimal distribution (S5 Appendix).

## Results

### The capacity-cost functions

We evaluated the information transmission capabilities for different stimulation scenarios distinguished by the amount of inhibition associated with the stimulus. In each scenario, the frequency of excitatory synapses ranged from $\lambda_{\text{exc}}^{\left(\text{bcg}\right)}$ to approximately $40\cdot \lambda_{\text{exc}}^{\left(\text{bcg}\right)}$, therefore the intensity of the stimulus can be represented by *A* ∈ [1, 40]:

$$\begin{array}{c} \lambda_{\text{exc}}=A\cdot \lambda_{\text{exc}}^{\left(\text{bcg}\right)}. \end{array}$$ (23)

The frequency of inhibitory synapses added on top of $\lambda_{\text{inh}}^{\left(\text{bcg}\right)}$ generally scales linearly with the intensity added on top of $\lambda_{\text{exc}}^{\left(\text{bcg}\right)}$, i.e. with *A* − 1. The frequency of inhibitory synapses can be than expressed with an *inhibition scaling factor* *B* as

$$\begin{array}{c} \lambda_{\text{inh}}=\lambda_{\text{inh}}^{\left(\text{bcg}\right)}(1+B(A-1)). \end{array}$$ (24)

**Fig 2:**
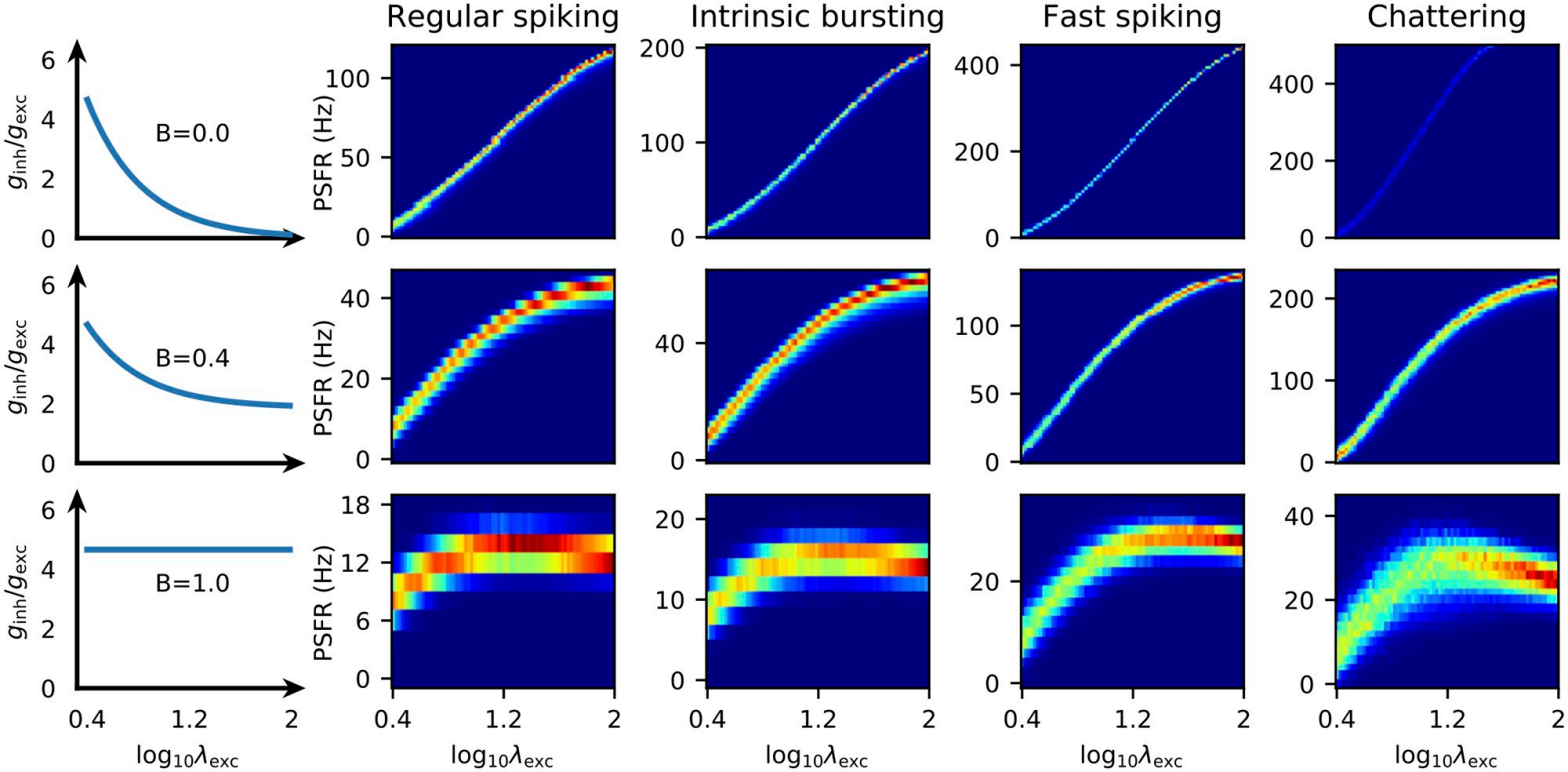
Stimulus-response relationships. Stimulus-response relationships for the MAT neurons specified by the parameters in Table A in S1 Appendix. Each row corresponds to a different inhibition regime. The ratio of inhibitory to excitatory conductance as a function of stimulus intensity is displayed in the leftmost column. The time window Δ was in this case chosen as 500 ms. The *x*-axis is logarithm of the rate of bombardment by excitatory synapses (Eq 23). The *y*-axis shows the post-synaptic firing rate (Eq 21). The rate of inhibitory synapses is specified by *B* (Eq 24). This Figure is also available with equally scaled *y*-axes for all neurons and regimes (S1 Fig).

**Fig 3:**
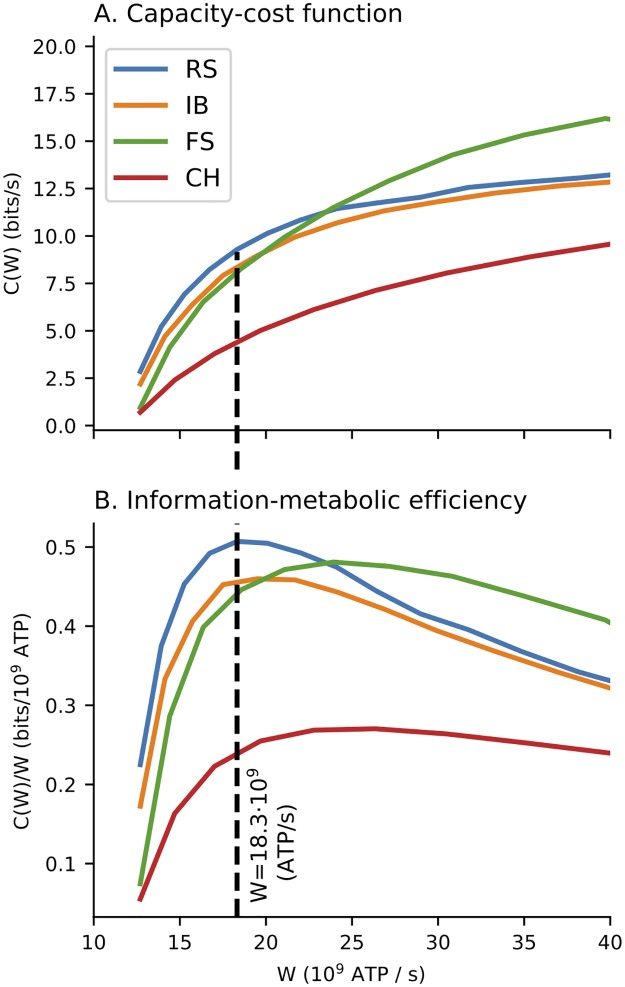
Capacity-cost function. Capacity-cost function (panel A) and capacity per spike (panel B) for the case of coding time window Δ = 100 ms and inhibition scaling factor *B* = 0.4. The dashed vertical line indicates the cost at which the optimal capacity per spike for the RS neuron is reached.

From the stimulus-response relationships (Fig 2) it is obvious that the fast spiking (FS) and chattering (CH) neurons have an advantage of a wide range of possible outputs. Also the excitation-only stimulation scenario (*B* = 0) allows for higher firing rates (i.e., offers wider coding range). However, when the metabolic expenses are taken into account the range of possible outputs becomes less important (because of the high associated expenses). This can be seen in Fig 3 where the capacity cost function for four different parameter sets of the MAT model (Table A in S1 Appendix) is shown and it is illustrated how the capacity cost function translates to bits per spike. The RS neuron is generally the most efficient independently either of the inhibition scaling factor *B* or the coding time window. Moreover, since at any allowed cost either the RS of the FS neuron offer the highest amount of transmitted information, we conclude that the bursting behavior is not beneficial for rate coding. This was also observed experimentally for temporal code [59].

### Inhibition stabilizes the membrane potential

We observed that higher inhibition to excitation ratios leads to lower membrane potential fluctuations. This arises as an effect of synaptic filtering and reversal potentials, which are both biologically important parts of neural communication and essential for observation of this phenomenon (see S4 Appendix for details). In [60], similar effect was reported for a membrane potential model without synaptic filtering, however, only for a strongly hyperpolarized membrane. The suppression of membrane potential fluctuations has also been observed in vivo [61].

**Fig 4:**
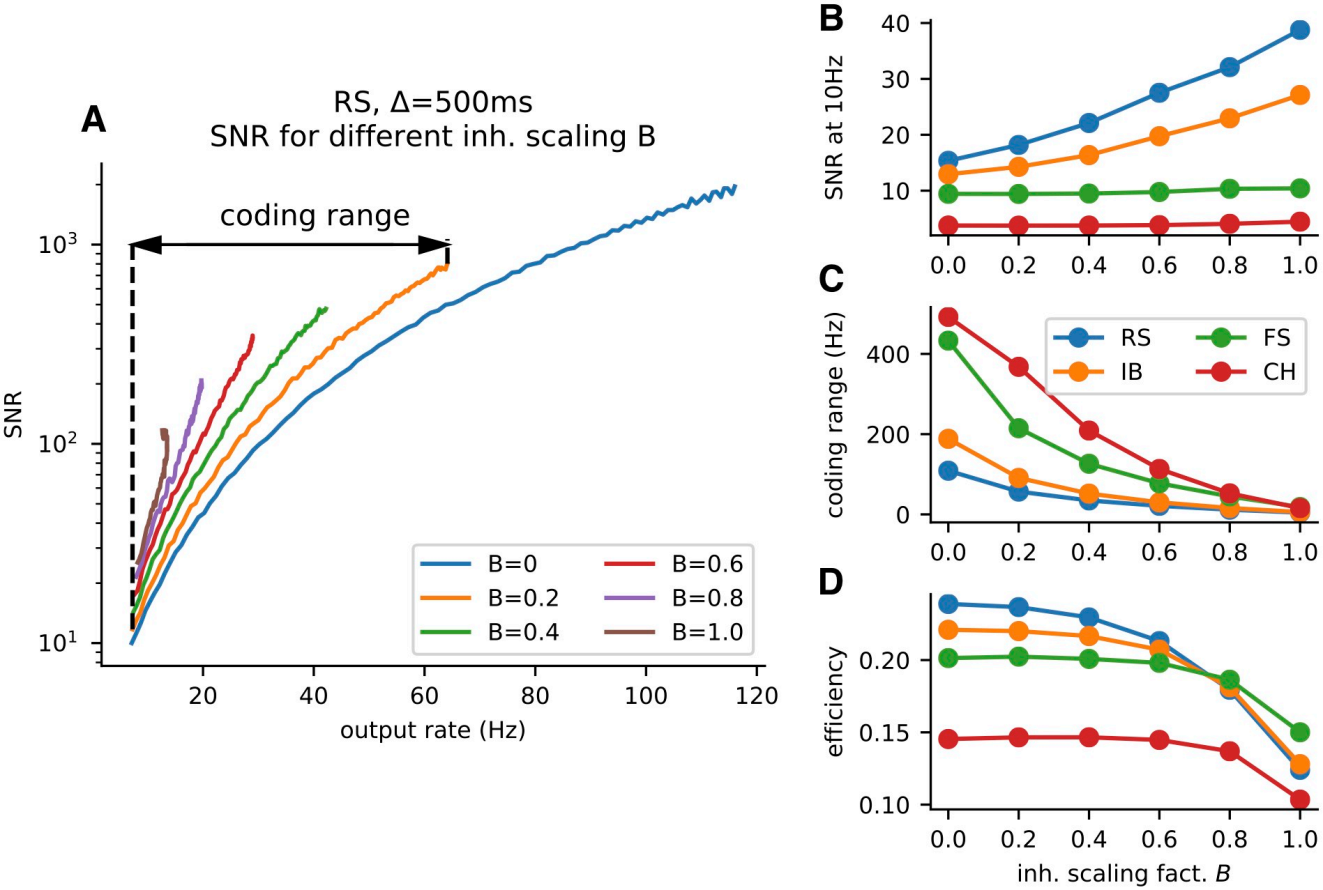
The effect of inhibition on metabolically efficient information transfer. (A) Signal-to-noise ratio (SNR, Eq (25)) of the RS neuron’s response as a function of the mean post-synaptic firing rate *r*(*x*) (Eq 26). Higher inhibition leads to a higher SNR, however, also to a lower coding range. The coding range for *B* = 0.2 is visualized in the plot. (B) The SNR at 10 Hz at different inhibition levels for all four neurons. The effect of the decreased membrane potential fluctuations on the FS and CH neurons is negligible, as opposed to the RS and IB neurons. (C) Decrease of the coding range with inhibition. (D) The metabolic efficiency in bits per spike (Eq (18)). The initial increase in the efficiency is almost negligible, however, the drop for *B* = 1 caused by the narrow coding range is apparent. The time window used for this figure is Δ = 500 ms.

The decrease in the membrane potential’s standard deviation leads to a more reliable firing rate (response) and subsequently higher signat-to-noise ratio (SNR) in regimes with stronger inhibition (Fig 4). For given time window Δ and inhibition scaling factor *B*, SNR is defined as

$$\begin{array}{c} \text{SNR}\left(x;\Delta ,B\right)=(\frac{r(x;\Delta ,B)}{s(x;\Delta ,B)}{)}^{2}, \end{array}$$ (25)

where *r*(*x*; Δ, *B*) is the mean response to the stimulus *x*, given the time window Δ and the inhibition scaling factor *B*, *s*(*x*; Δ, *B*) is the standard deviation of the response:

$$r(x)=\frac{1}{\Delta }\sum_{y=0}^{+\infty }yf\left(y|x\right),$$ (26)

$$s(x)=\frac{1}{\Delta }\sqrt{\sum_{y=0}^{+\infty }y^{2}f\left(y|x\right)-r{\left(x\right)}^{2}}.$$ (27)

### The effect of inhibition on metabolic efficiency

**Fig 5:**
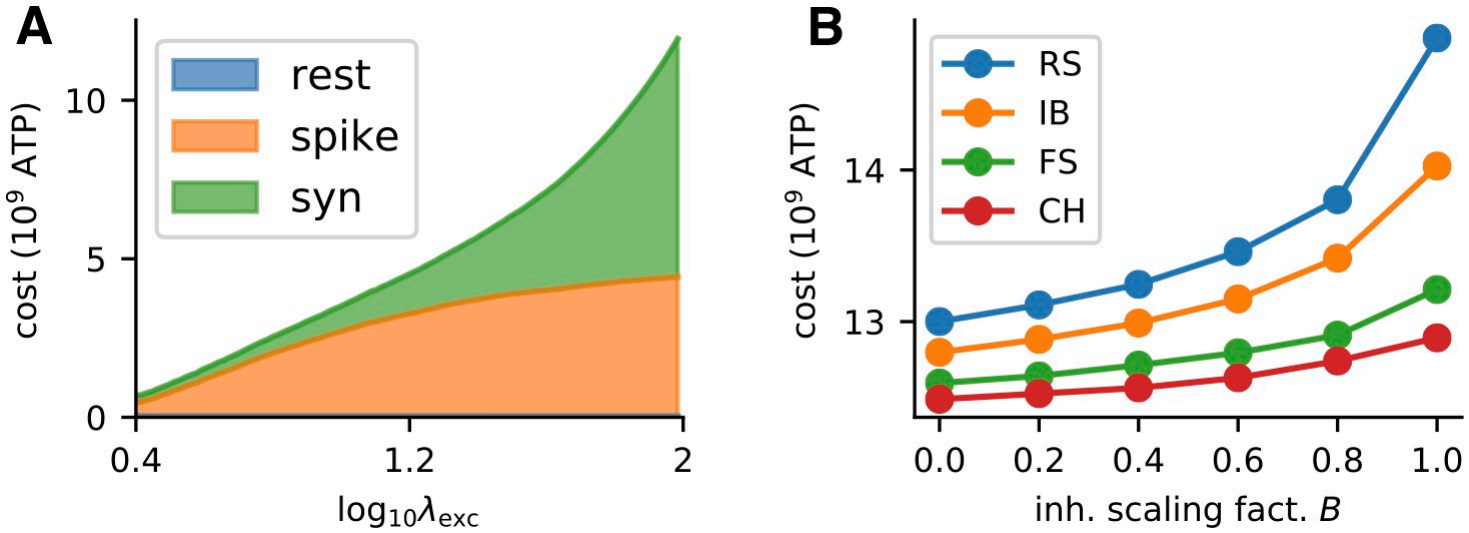
Metabolic cost of neural activity. (A) Cost of response for a given input *x* = λ_exc_, RS neuron (Tab A in S1 Appendix), Δ = 100 ms, *B* = 0.4. (B) Cost of maintaining a firing rate of 12 Hz for 100 ms for different values of inhibition to excitation ratio.

The higher ratio of inhibition to excitation also has some negative consequences:

1. The inhibition limits the possible depolarization of the membrane and the neuron is unable to attain high firing rates. We quantify this by defining the coding range:
  $$\begin{array}{c} \text{CR}\left(\Delta ,B\right)=\max_{x_{1},x_{2}}(r\left(x_{2};\Delta ,B\right)-r\left(x_{1};\Delta ,B\right)). \end{array}$$ (28)
  We observe that the coding range is generally decreased with increased amount of inhibition (Figs 2 and 4A).
2. To attain identical mean firing rate with higher excitation to inhibition ratio, the excitatory synaptic current has to be larger and therefore such stimulation is associated with higher metabolic costs (Fig 5).

Surprisingly, the information theoretical efficiency is generally unaffected by the level of inhibition, meaning that the increase in signal to noise ratio and decrease of coding range effectively even out. This holds up to a certain point, when the coding range becomes too narrow and the efficiency of information transfer starts dropping dramatically (Fig 4D).

### The optimal PSFR histograms

**Fig 6:**
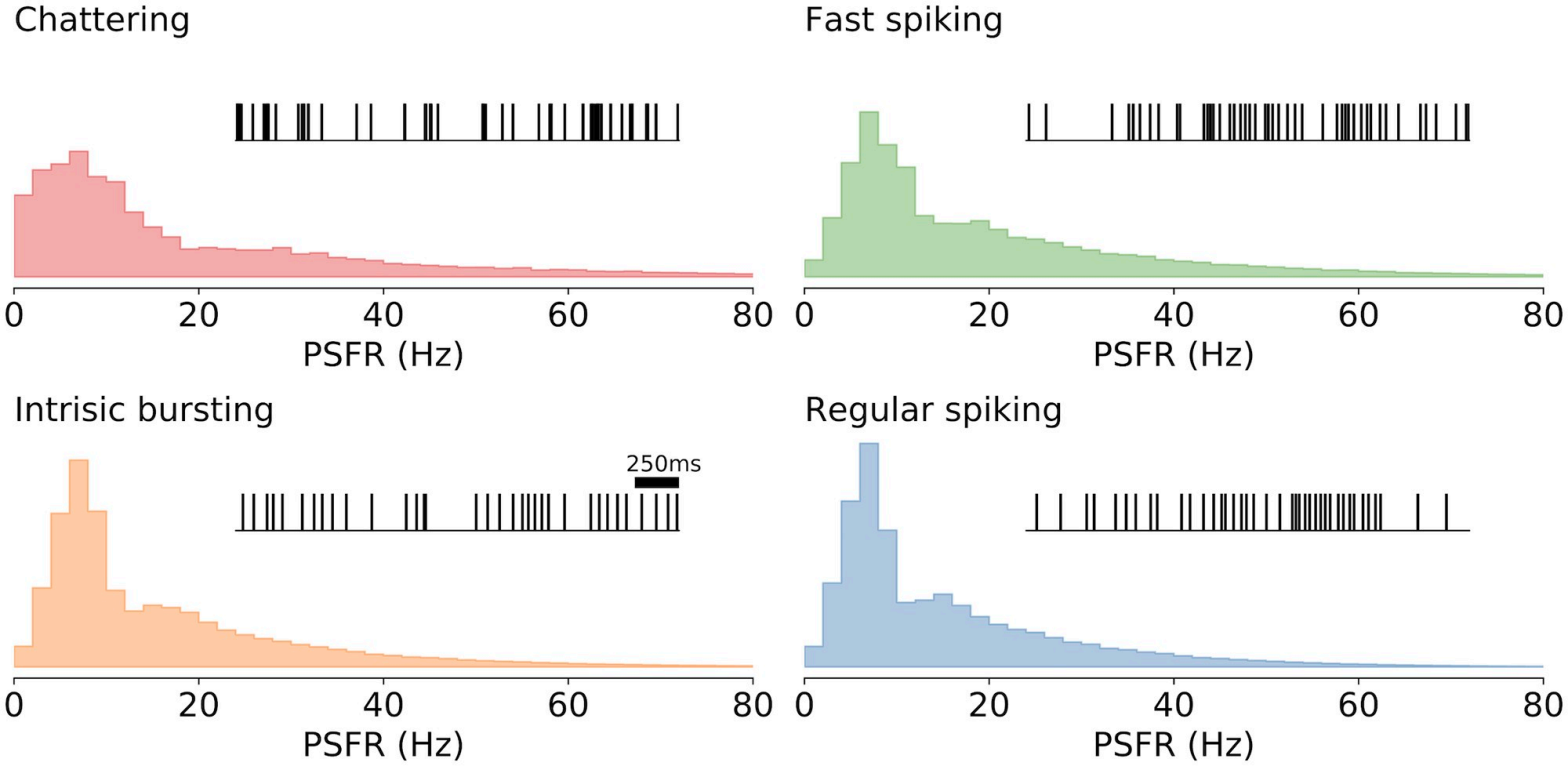
Predicted PSFR histograms. Post-synaptic firing rate histograms corresponding to the metabolically efficient regime with the coding time window Δ = 500 ms and inhibition scaling factor *B* = 0.4 for the four different neurons. Unlike the statistics of the input, the output statistics can be measured in vivo and can therefore be used to verify whether a neuron employs metabolically efficient coding. A typical spike train in the efficient regime is shown for each neuron.

By evaluating the information-metabolic efficiency we also obtain the optimal input-output statistics. The resulting optimal post-synaptic firing rate (PSFR) histograms (Eq (11)) provide a potentially testable prediction (Fig 6). Our predictions need to be tested against long in-vivo recordings, such as in [33, 62, 63]. Qualitatively, our predictions agree with the observations in [33], that the probabilities of large firing rates are suppressed, moreover, the tail is approximately exponential with respect to the metabolic cost (Eq 10), as observed by Polavieja [30, 31]. Polavieja assumes that the overall cost grows linearly with the output rate. For the case of metabolic cost considered in this paper, the nonlinearity is important mostly for high firing rates.

### Optimal input distributions

**Fig 7:**
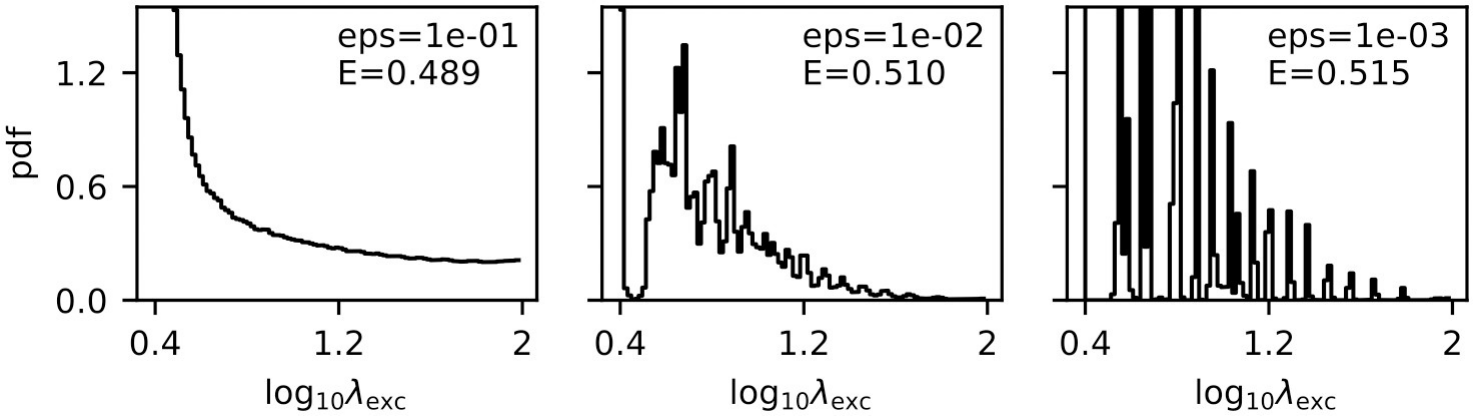
Approximately optimal input probability distributions. The plots show different input probability distributions obtained from different steps of the Jimbo-Kunisawa algorithm. For each input distribution the estimated efficiency *E* (in bits / 10^9^ ATP) is given in the plot together with the relative error eps (to the true value of the efficiency). The true value of the efficiency (Eq (18)) can be nearly reached with very different input probability distributions.

As we showed in the Methods section, the optimal input distribution has non-zero probability only for a finite number of points. However, the optimal conditions can be nearly reached by many different input distributions (Fig 7). Generally, we see a trend towards more pronounced discreteness if we desire to be closer to the true optimum. However, the increases in efficiency and effect on the PSFR distribution are only marginal. Therefore, unlike in the case of PSFR distribution which is robust, the optimal input distribution is difficult to relate to real data.

Yet we can observe that in the metabolically efficient regime, significant portion of the probability is given to the weakest input, i.e., purely spontaneous activity. For a population of independently encoding neurons this would mean that at any moment, most of them would be exhibiting only spontaneous activity.

### Rate coding time-scale

**Fig 8:**
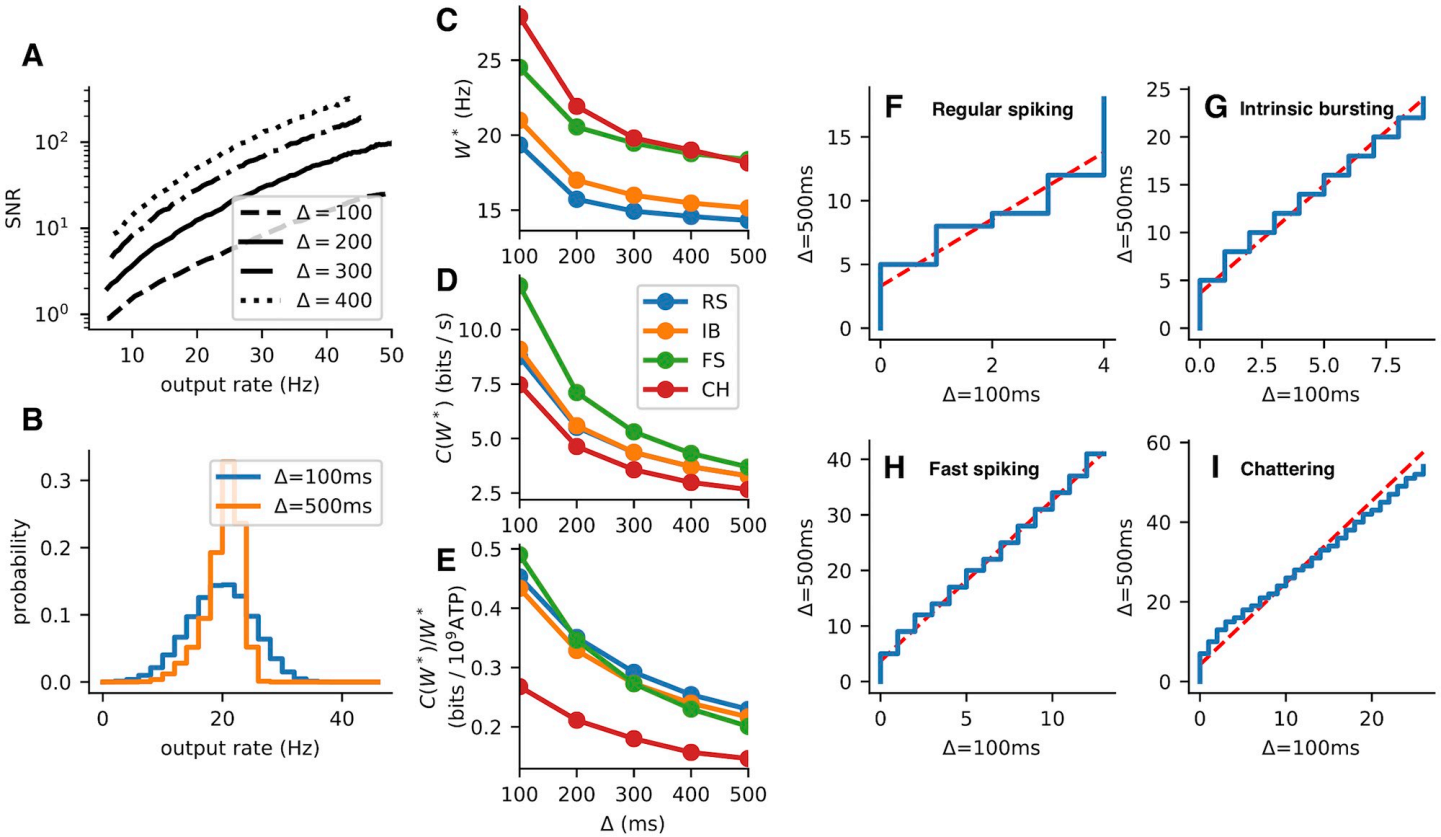
The effect of coding time window on metabolically efficient information transmission. (A) Signal to noise ratio for different coding time windows as a function of the mean response *r*(*x*) (Eq 26). (B) Comparison of response to a given stimulus (producing a response rate of approx 20 Hz) for different coding time windows. In order to get comparable results, the distribution on number of spikes in 100 was five times convoluted with itself. The distribution for 100 ms is more spread due to the adaptation effects. (C) Optimal mean PSFR (Eq 22). (D) Information capacity with the optimal metabolic expenses. (E) Metabolic efficiency in bits per spikes. The decrease with the length of the coding time window shows us, that the adaptation effects visible in B don’t play a significant role in this case. (F-I): Quantile-quantile plots comparing the PSFR distributions for different coding time windows. The red dashed line is a linear fit, acting as a visual guide. In the case of metabolically-efficient coding invariant on time scale, the q-q plots shouldn’t deviate significantly from the line. This holds for the RS and FS neurons, for the most part also for the IB neuron. For all plots in the figure the inhibition scaling factor *B* = 0.4 was used.

Naturally, longer time windows will lead to a higher signal to noise ratio (Eq (25))—we will be better able to identify a stimulus if we “listen” longer (Fig 8A). For a truly memoryless channel, however, the use of a shorter time window must always result in higher information capacity (measured in bits per second). Mutual information from two subsequent uses of a memoryless channel (with inputs **x** = {*x*_1_, *x*_2_} and outputs **y** = {*y*_1_, *y*_2_}) is always lower or equal than double of the mutual information resulting from a single use [64]:

$$\begin{array}{c} I\left(x;y\right)=2I\left(x_{1};y_{1}\right)-I\left(y_{1};y_{2}\right), \end{array}$$ (29)

*I*(*y*_1_; *y*_2_) being maximal for extreme correlation between the inputs, i.e. *x*_1_ = *x*_2_. Moreover, *I*({*x*_1_, *x*_2_}; *y*_1_ + *y*_2_) < *I*({*x*_1_, *x*_1_}; {*y*_1_, *y*_2_}), since we are losing information about the temporal structure of the response. Therefore, given any probability distribution of the inputs, the mutual information for channel with a half-sized coding time window will always be higher (in bits per second):

$$\begin{array}{c} I\left(\left\{x_{1},x_{1}\right\};y_{1}+y_{2}\right)<I\left(\left\{x_{1},x_{1}\right\};\left\{y_{1},y_{2}\right\}\right)<2I\left(x_{1};y_{1}\right). \end{array}$$ (30)

In our case, the neurons are not truly memoryless channels. They exhibit adaptation, which we took into consideration in the optimization process by using an algorithm we developed specifically for this purpose (S5 Appendix). Due to adaptation, the number of spikes is influenced by the previous stimulus, thus additional noise to the stimulus-response relationship is introduced. We illustrate this by comparing the PSFR histogram for a given stimulus intensity and a coding time window Δ = 500 ms with the PSFR histogram for a coding time window Δ = 100 ms, five times convoluted with itself, corresponding to and equal mean PSFR (Fig 8B). For a memoryless channel, the distributions would be identical. However, the distribution obtained by using a shorter time window is more spread.

We observe that while the length of time window doesn’t significantly influence the mean PSFR, the information capacity with the optimal mean PSFR drops and so does the associated efficiency in bits per spike (Fig 8C–8E). Therefore we can conclude the adaptation effects aren’t significant enough to make coding on longer time scales more beneficial. Interestingly, however, not only the mean PSFR do not seem to be much affected by the length of the coding time window (Fig 8C), but also the shape of the PSFR histogram (computed from the optimal input distribution by Eq (11)) seems to be rather unaffected by the length of the coding time window (Fig 8F–8I).

### The effect of model parameters and spontaneous firing rate

In order to provide a meaningful comparison of different firing patterns, we have so far considered such parameters of the MAT model that lead to an approximately equal spontaneous firing rate (by spontaneous firing rate we mean the average response to the background noise, specified in S3 Appendix). However, it is known that neurons across different layers of the cortex exhibit different spontaneous firing rates (e.g., [65–67]).

To calculate the spontaneous activity we take advantage of the approximate formula describing the stationary firing rate *f* of MAT model if stimulated with a constant current *I* [68]:

$$\begin{array}{c} f≐\frac{1}{\tau_{2}\;\text{log}\;(\frac{\alpha_{2}}{IR-\omega }+1)}. \end{array}$$ (31)

**Fig 9:**
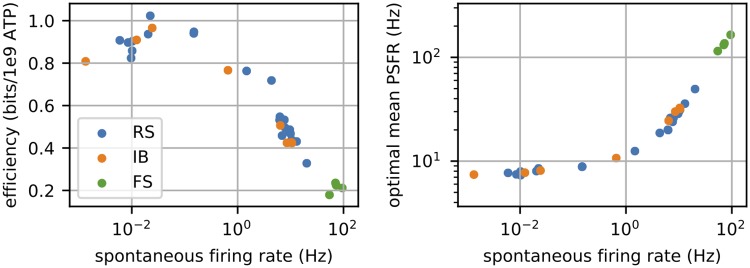
Cortical neurons. The *x*-axis in both graphs is the spontaneous firing rate of the 34 neuronal models corresponding to the cortical neurons, i.e., their response to the simulated background noise. The information-metabolic efficiency (Eq (18)) and optimal mean PSFR (Eq (22)) was calculated for the case of constant inhibition (*B* = 0, Δ = 100 ms).

In order to gain a general insight into the dependence of the predictions on the model parameters, we calculated the predicted mean PSFR (Eq (22)) and efficiency (Eq (18)) for 34 parameter sets corresponding to 34 neurons from the layers 2/3 and 5 of the rat motor cortex (used in [40]), kindly provided by Prof. Kobayashi. As expected, both efficiency and the optimal mean PSFR are strongly related to the spontaneous firing rate (Fig 9).

We confirmed that Eq (31) can be utilized to predict the spontaneous firing rate (see S6 Appendix for details) and therefore we conclude that the spontaneous firing rate and consequently also the information-metabolic efficiency are governed predominantly by *α*_2_ and *ω*. Moreover, increase in any of the two parameters leads to an increase in the spontaneous firing rate and therefore increase in the mean optimal PSFR and decrease in the information-metabolic efficiency.

## Discussion

The information capacity tells us what is the maximal amount of information a neuron could potentially reliably transfer. It is, however, beyond the scope of this work to investigate whether neurons utilize their full capacity and if so, how [34, 69]. The efficient coding hypothesis [2] leads us to believe that neurons are in some sense optimal. They need to transfer information fast and reliably and minimize the metabolic costs at the same time. This paper uses the value of information capacity per spike to take into account both information transmission and metabolic costs. Maximization of the information capacity per metabolic expenses leads to suppression of high post-synaptic firing rates observed in in-vivo recordings [33].

Analyses of this type generally have to rely on number of assumptions, including the nature of the input and the coding time scale. To mimic the nature of real neuronal synapses, we consider excitatory and inhibitory input with reversal potentials. The typical approach is to model the excitatory and inhibitory conductances as an Ornstein-Uhlenbeck process [34, 40, 49, 70], however, it has been shown that for consistency reasons, modeling the input as a shot noise with an exponential envelope is more appropriate [71]. We recreate the effect of the background network activity, during which the excitatory and inhibitory synaptic currents seem to be approximately balanced [72–77]. We then systematically explore several different input regimes differing in the amount of inhibition accompanying excitation during stimulation. This allows us to compare the different regimes by their information-energetic efficiency. Such systematic exploration also allows us to make less assumptions about the actual nature of the neuronal input and the results can also provide insight into what kind of dependency between excitation and inhibition is optimal.

The MAT model is remotely related to the model analyzed by Suksompong et. al. [36], where the threshold function can be generalized to behave similarly to MAT model. However, the key differences are in the assumptions on encoding (in [36] the information is assumed to be encoded in a sequence of inter-spike intervals, whereas we consider the rate coding) and in the input.

If the investigated neuronal model exhibits adaptation to the stimulus (as e.g. the MAT model does), the coding time scale is typically significantly limited from below, so that the influence of previous stimuli on the current response is negligible. We try to overcome this issue by proposing an algorithm which partially takes into account the effect of the previous stimulus. This is an important part of the optimization process, because otherwise we could overestimate the benefits of inhibition on the information-metabolic efficiency (Fig A in S5 Appendix).

The comparison of different noise levels was inspired by the work [70], where it was suggested that balanced excitatory and inhibitory currents lead to more efficient information transfer. Our results can’t be compared straightforwardly with [70] for several reasons. In our work, the state with balanced excitatory and inhibitory currents was considered to be the base state and we were investigating different regimes of stimulation of such neuron, whereas the work of Sengupta et. al. [70] focuses on the benefits of the balanced state. Moreover, in [70] the direct method [17] was used for evaluating information, which measures the entropy of spike trains without any reference to the stimuli, whereas we were investigating the information-transmission properties with the assumption that the neurons use rate code and computed the information capacity [10, 22] to evaluate the limits of information transmission. We observed a positive effect of higher inhibition, however, in the investigated stimulation scenarios the overall information efficiencies in bits per spike were largely unaffected by the inhibitory presynaptic activity. Robustness of the information-metabolically optimal properties with respect to the change of amount of inhibition in the system has also been recently reported by Harris et al. [78].

Numerically, our results are consistent with, e.g., [34], with the information efficiency being in the order of 0.1 bits per 10^9^ ATP molecules expended. Despite the differences in spiking patterns among the neuronal classes (RS, IB, FS, CH), as quantified by local variability [79], we find that the information metabolic efficiency of the rate code is mainly governed by neuronal spontaneous activity.

We considered both the excitatory and inhibitory rates (added on top of the modulatory background network activity) to scale linearly with the stimulus intensity, since this is the simplest scenario that can be considered. For most of the stimulation scenarios, we did not observe a significant change in the information-metabolic efficiency, however, if the inhibitory rates scaled slower than linearly, we could achieve both high signal to noise ratio and a wide coding range. Such regime is likely to employ very high rates of synaptic bombardment, therefore in such case one should also consider the cost of the pre-synaptic activity.

Our results deal with a single neuron, in accord with most of the previously published work [25]. Nevertheless, Eqs (15) or (17) are easily extendable to the case of a simple homogeneous population [80]. One may also investigate the multidimensional stimulus-response relationship in a group of coupled neurons, however, the corresponding optimization is performed over joint probability distributions which becomes quickly untractable as the population size grows. It is also worth noting that the problem of optimal information transmission through nodes in general networks is still open and Eq (15) might not be directly useful [81].

To summarize the results of our work as follows:

- By employing a novel method for calculating the information transmission capabilities of channels displaying adaptation to the stimulus (S5 Appendix) we calculated numerically the information transmission capabilities of the MAT model [40] for biologically relevant parameters under metabolic constraints on different time scales and with different levels of inhibition.
- We used the results of Richardson [71] to show that inhibition can stabilize the membrane potential, leading to a more reliable response of the MAT model. To the best of our knowledge, this counter-intuitive effect of inhibition, for which we provide a theoretical justification, has not yet been reported.
- We found that the regular spiking (RS) neuron offers best information transmission per single spike, but when more energy is available, more information can be transmitted by the behavior common to fast spiking (FS) neurons. Neurons exhibiting the bursting behavior (IB, CH) were shown not to be very effective for rate coding in the investigated regimes.
- Due to adaptation effects shorter rate coding time windows led to lower signal to noise ratios. Despite the increase in noise, information can be transferred more efficiently with shorter time windows. However, we observed that the length of the time window does not significantly affect the shape of the PSFR histograms, which have the potential to be compared to experimental data.
- We found that the metabolic efficiency is surprisingly robust towards the changes in the amount of inhibition accompanying the excitation. Moreover, we observed that increased inhibition leads to higher signal to noise ratio, but also to a drop in the coding range. This does not affect the metabolic efficiency significantly until a certain point, when the coding range is so narrow that information cannot be transferred efficiently by rate code.
- We pointed out that the optimal input for a neuron using rate code has non-zero probability only for a finite number of inputs. However, by showing different input distribution, which nearly achieve the information-metabolic efficiency, we illustrated that the discreteness of the input is not a necessary condition for an effective communication.

The core of the simulation code was written in C++ and packaged as a Python module using Cython. This module is available on GitHub (https://github.com/Tom83B/matsim). The analysis of the data was done in Python using the NumPy and SciPy libraries. All necessary code was also uploaded to a GitHub repository (https://github.com/Tom83B/rate-code-eff-2019).

## Supporting information

S1 Appendix Parameters of the MAT model. (PDF)

S2 Appendix Numerical simulation of the leaky integrator. (PDF)

S3 Appendix Spontaneous activity. (PDF)

S4 Appendix Variability of the steady-state membrane potential. (PDF)

S5 Appendix Information capacity of channels exhibiting adaptation. (PDF)

S6 Appendix Approximation of spontaneous firing rate. (PDF)

S1 Fig Stimulus-response relationships with identical scales. Same as Fig 2, but the scales are same for all the neurons and inhibition scaling factors *B*. Each row corresponds to a different inhibition regime. The ratio of inhibitory to excitatory conductance as a function of stimulus intensity is displayed in the leftmost column. The time window Δ was in this case chosen as 500 ms. The *x*-axis is logarithm of the rate of bombardment by excitatory synapses (Eq 23). The *y*-axis shows the post-synaptic firing rate (Eq 21).
(PDF)
