# Supplementary material for: The effect of inhibition on rate code efficiency indicators
Source: PLoS Comput Biol. 2019 Dec 2;15(12):e1007545. doi: 10.1371/journal.pcbi.1007545 (PMC6907877; doi:10.1371/journal.pcbi.1007545)
Supplement: S1 Appendix — (PDF) [file pcbi.1007545.s001.pdf]

Table A: Parameters of studied neurons

	α_1	α_2	ω
Regular spiking (RS)	$30\,\mathrm{mV}$	$2.0\,\mathrm{mV}$	$-65\,\mathrm{mV}$
Intrinsive bursting (IB)	$7.5\,\mathrm{mV}$	$1.5\,\mathrm{mV}$	$-64.3\,\mathrm{mV}$
Fast spiking (FS)	$10\,\mathrm{mV}$	$0.2\,\mathrm{mV}$	$-62.4\,\mathrm{mV}$
Chattering (CH)	$-0.5\,\mathrm{mV}$	$0.4\,\mathrm{mV}$	$-61.8\,\mathrm{mV}$

S1 Appendix

Parameters of the MAT model.

Kobayashi et al. (2009) identified sets of parameters of the MAT model that correspond to real cortical neurons belonging to different classes, based on their spiking pattern (FS, IB, RS) or are capable of reproducing the same general behavior (CH). The following parameters were common for all four neurons: $\tau_\mathrm{m} = 5\,\mathrm{ms}$, $R = 50\,\mathrm{M\Omega}$ and $\tau_1 = 10\,\mathrm{ms}$, $\tau_2 = 200\,\mathrm{ms}$, $L = 2$. We used the values of α_1 and α_2 as suggested in Kobayashi et al. (2009) and chose the value of ω so that the mean firing rate in the presence of spontaneous background activity (i.e. $\lambda_\mathrm{exc} = \lambda_\mathrm{exc}^{(\mathrm{bcg})}$, $\lambda_\mathrm{inh} = \lambda_\mathrm{inh}^{(\mathrm{bcg})}$) was approximately $8\,\mathrm{Hz}$ in the steady state. This is roughly in accordance with spontaneous firing rates observed in awake animals (e.g., Steriade et al. (2001); O'Connor et al. (2010)). The exact value of spontaneous firing rate is not crucial, since it does not affect the qualitative character of the results. The used values of the free parameters α_1, α_2 and ω are given in Table A.

References

R. Kobayashi, Y. Tsubo, and S. Shinomoto. Made-to-order spiking neuron model equipped with a multi-timescale adaptive threshold. *Front Comput Neurosci*, 3:9, 2009.

D. H. O'Connor, S. P. Peron, D. Huber, and K. Svoboda. Neural activity in barrel cortex underlying vibrissa-based object localization in mice. *Neuron*, 67(6):1048–1061, sep 2010. doi: 10.1016/j.neuron.2010.08.026.

M. Steriade, I. Timofeev, and F. Grenier. Natural waking and sleep states: A view from inside neocortical neurons. *Journal of Neurophysiology*, 85(5): 1969–1985, may 2001. doi: 10.1152/jn.2001.85.5.1969.
