# Supplementary material for: The effect of inhibition on rate code efficiency indicators
Source: PLoS Comput Biol. 2019 Dec 2;15(12):e1007545. doi: 10.1371/journal.pcbi.1007545 (PMC6907877; doi:10.1371/journal.pcbi.1007545)
Supplement: S2 Appendix — (PDF) [file pcbi.1007545.s002.pdf]

S2 Appendix.

Numerical simulation of the leaky integrator.

Eq (1) may be rewritten as:

$$\frac{\mathrm{d}V}{\mathrm{d}t} = -\frac{1}{\tau(g_\mathrm{e}, g_\mathrm{i})}\left(V - \hat{V}(g_\mathrm{e}, g_\mathrm{i})\right), \tag{S2.1}$$

where

$$\tau(g_\mathrm{e}, g_\mathrm{i}) = \frac{\tau_m}{1 + R(g_\mathrm{e} + g_\mathrm{i})}, \tag{S2.2}$$

$$\hat{V} = \frac{E_\mathrm{L} + R(g_\mathrm{e}E_\mathrm{e} + g_\mathrm{i}E_\mathrm{i})}{1 + R(g_\mathrm{e} + g_\mathrm{i})}. \tag{S2.3}$$

Considering the conductances to be constant during the integration time step $\Delta t = 0.1\,\mathrm{ms}$, we can integrate the equation as follows:

$$V_{n+1} = \hat{V} + (V_n - \hat{V})\exp(-\Delta t/\tau). \tag{S2.4}$$
