# Supplementary material for: The effect of inhibition on rate code efficiency indicators
Source: PLoS Comput Biol. 2019 Dec 2;15(12):e1007545. doi: 10.1371/journal.pcbi.1007545 (PMC6907877; doi:10.1371/journal.pcbi.1007545)
Supplement: S3 Appendix — (PDF) [file pcbi.1007545.s003.pdf]

S3 Appendix.

Spontaneous activity.

In (Destexhe et al., 2001) the values of average and standard deviation of the conductances during spontaneous background activity were reported as 0.012 and 0.0030 µS for g_{exc} and 0.057 and 0.0066 µS for g_{inh}. The goal was to reproduce these values using shot noise with exponential decay described by Eqs (5,6).

When the spike arrival times are Poissonian, the following holds (e.g., Rajdl and Lansky (2015)):

$$\mathrm{E}(g_{\mathrm{exc}}(t)) = \tau_{\mathrm{exc}}\bar{g}_{\mathrm{exc}}\lambda_{\mathrm{exc}}, \tag{S3.1}$$

$$\mathrm{Var}(g_{\mathrm{exc}}(t)) = \frac{1}{2}\tau_{\mathrm{exc}}\lambda_{\mathrm{exc}}\bar{g}^2_{\mathrm{exc}}. \tag{S3.2}$$

Then it follows, that for fixed τ_{exc} and mean and variance given, the intensity and peak conductance have to be chosen as

$$\bar{g}_{\mathrm{exc}} = 2\frac{\mathrm{Var}(\mathrm{g}_{\mathrm{exc}}(\mathrm{t}))}{\mathrm{E}(\mathrm{g}_{\mathrm{exc}}(\mathrm{t}))} \tag{S3.3}$$

$$\lambda_{\mathrm{exc}} = \frac{\mathrm{E}(\mathrm{g}_{\mathrm{exc}}(\mathrm{t}))}{\bar{g}_{\mathrm{exc}}\tau_{\mathrm{exc}}}. \tag{S3.4}$$

Computation of $\bar{g}_{\mathrm{exc}}$ and λ_{exc} is then identical.

References

A. Destexhe, M. Rudolph, J. M. Fellous, and T. J. Sejnowski. Fluctuating synaptic conductances recreate in vivo-like activity in neocortical neurons. *Neuroscience*, 107:13–24, 2001. ISSN 0306-4522.

K. Rajdl and P. Lansky. Stein's neuronal model with pooled renewal input. *Biological Cybernetics*, 109(3):389–399, apr 2015. doi: 10.1007/s00422-015-0650-x.
