# Supplementary material for: The effect of inhibition on rate code efficiency indicators
Source: PLoS Comput Biol. 2019 Dec 2;15(12):e1007545. doi: 10.1371/journal.pcbi.1007545 (PMC6907877; doi:10.1371/journal.pcbi.1007545)
Supplement: S4 Appendix — (PDF) [file pcbi.1007545.s004.pdf]

S4 Appendix.

Variability of the steady-state membrane potential.

Mean and variance of the membrane potential (given by Eq (1)), driven by Poissonian shot noise conductances, can be approximated as (Richardson and Gerstner, 2005):

$$\langle V \rangle = \mu_V + E_0 \tag{S4.1}$$

$$\langle V^2 \rangle - \langle V \rangle^2 = \left(\frac{\sigma_{\text{exc}}}{g_0}\right)^2 \mathcal{E}_{\text{exc}}^2 \frac{\tau_{\text{exc}}}{\tau_{\text{exc}} + \tau_0} + \left(\frac{\sigma_{\text{inh}}}{g_0}\right)^2 \mathcal{E}_{\text{inh}}^2 \frac{\tau_{\text{inh}}}{\tau_{\text{inh}} + \tau_0}, \tag{S4.2}$$

where σ_{exc}^2, σ_{inh}^2 are the variances of the shot noise (Eq (S3.2)) and

$$g_0 = \frac{1}{R} + g_{\text{exc0}} + g_{\text{inh0}}, \tag{S4.3}$$

$$E_0 = \frac{1}{g_0}\left(\frac{1}{R}E_L + g_{\text{exc0}}E_{\text{exc}} + g_{\text{inh0}}E_{\text{inh}}\right), \tag{S4.4}$$

$$\mathcal{E}_{\text{exc}} = E_{\text{exc}} - E_0, \tag{S4.5}$$

$$\mathcal{E}_{\text{inh}} = E_{\text{inh}} - E_0, \tag{S4.6}$$

$$\tau_0 = \frac{\tau_m}{R g_0}, \tag{S4.7}$$

$$\mu_V = \left(\frac{\sigma_{\text{exc}}}{g_0}\right)^2 \mathcal{E}_{\text{exc}} \frac{\tau_{\text{exc}}}{\tau_{\text{exc}} + \tau_0} + \left(\frac{\sigma_{\text{inh}}}{g_0}\right)^2 \mathcal{E}_{\text{inh}} \frac{\tau_{\text{inh}}}{\tau_{\text{inh}} + \tau_0} \tag{S4.8}$$

g_{exc0}, g_{inh0} denote the shot noise means (S3 Appendix):

$$g_{\text{exc0}} = \tau_{\text{exc}} \bar{g}_{\text{exc}} \lambda_{\text{exc}}, \tag{S4.9}$$

$$g_{\text{inh0}} = \tau_{\text{inh}} \bar{g}_{\text{inh}} \lambda_{\text{inh}}. \tag{S4.10}$$

These approximations provide a very good estimate of the mean and standard deviation of the membrane potential (Fig A). Both in the theoretical approximations and in the simulated values we see that given the same mean value of the membrane potential, with higher inhibition to excitation ratio, the standard deviation of the membrane potential drops. Some insight can be gained by analyzing the limit case, i.e., λ_{exc} and λ_{inh} both tending to infinity while the inhibition scaling factor B is held constant. Then:

$$\langle V \rangle \to \frac{\tau_{\text{exc}} \bar{g}_{\text{exc}} E_{\text{exc}} + B \tau_{\text{inh}} \bar{g}_{\text{inh}} E_{\text{inh}}}{\tau_{\text{exc}} \bar{g}_{\text{exc}} + B \tau_{\text{inh}} \bar{g}_{\text{inh}}}, \tag{S4.11}$$

$$\sigma_V = \sqrt{\langle V^2 \rangle - \langle V \rangle^2} \to 0. \tag{S4.12}$$

With increasing B, $\langle V \rangle$ drops and therefore, hypothetically, we can reach zero variance of the membrane potential and the lower the desired mean value, the higher B is needed.

Although we used the Poisson shot noise with an exponential filtering as an input, the results apply also for the Ornstein-Uhlenbeck input approximation. The only difference will be in Eq (S4.1), because for the Ornstein-Uhlenbeck approximation $\langle V \rangle = E_0$ (Richardson, 2004; Richardson and Gerstner, 2005).

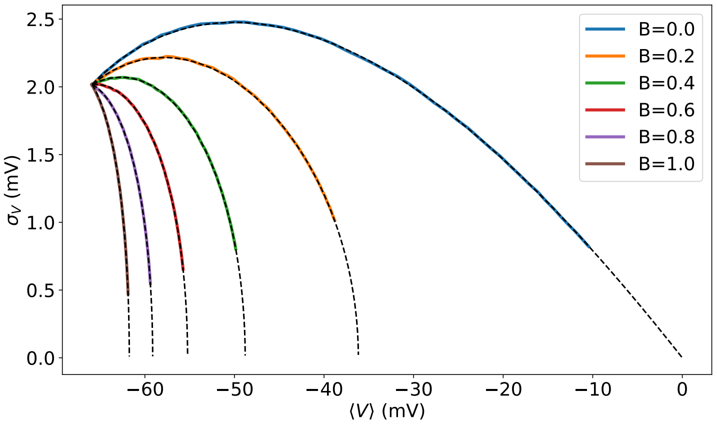

Figure A: Standard deviation of the membrane potential as a function of its mean. Color-coded are different inhibition to inhibition scaling factor B. Dashed lines indicate the theoretical approximations (Eqs (S4.1, S4.2))

References

M. J. E. Richardson. Effects of synaptic conductance on the voltage distribution and firing rate of spiking neurons. *Physical Review E*, 69(5), may 2004. doi: 10.1103/physreve.69.051918.

M. J. E. Richardson and W. Gerstner. Synaptic shot noise and conductance fluctuations affect the membrane voltage with equal significance. *Neural Computation*, 17(4):923–947, apr 2005. doi: 10.1162/0899766053429444.
