# Supplementary material for: The effect of inhibition on rate code efficiency indicators
Source: PLoS Comput Biol. 2019 Dec 2;15(12):e1007545. doi: 10.1371/journal.pcbi.1007545 (PMC6907877; doi:10.1371/journal.pcbi.1007545)
Supplement: S5 Appendix — (PDF) [file pcbi.1007545.s005.pdf]

S5 Appendix.

Information capacity of channels exhibiting adaptation.

Classical algorithm for computation of the information capacity C (Eq (15)) of a memoryless channel withou feedback and the optimal input distribution is the Blahut-Arimoto algorithm (Blahut, 1972). Blahut (1972) also offers an extension of the algorithm for computation of the constrained capacity $C(W)$ (Eq (17)), however, the cost restriction is not straightforward and we used the cutting plane algorithm (Huang and Meyn, 2005; Kelley, 1960) for computation of the capacity-cost function instead (for condensed summary of the algorithm in the context of neuroscience see (Kostal and Lansky, 2013, Appendix A)).

For evaluating the capacity-cost functions, is is necessary to evaluate $C(W)$ for many different values of the cost W. When only the optimal state (maximal amount of transferred information per unit of cost) is of interest, it is possible to use the Jimbo-Kunisawa algorithm Jimbo and Kunisawa (1979); Suksompong and Berger (2010), for which the input distribution converges towards the state of maximal $C(W)/W$.

For neurons, it takes some time for the neuron to reach its steady state. A transition between an intense stimulus to a less intense one is usually followed by a period of silence when no spike is fired, because the dynamic threshold takes some time to reach the new level of the membrane potential. On the other hand, transitions from a less intense stimulus to a more intense one causes an immediate increase in the firing rate which then slowly decreases until it reaches its steady state value. Thus the statistics of transitions between stimuli can have a significant effect on the observed response statistics to individual stimuli.

To mitigate the effect of past stimuli, ideally, we want the stimulus response relationship to be generated with the same input statistics as the S-R relationship predicts to be the optimum. However, the optimal input statistics depend on the S-R relationship. We solved the problem in the following manner:

1. For each possible stimulus s, we evaluate the stimulus response relationship $P_s(y|x)$ - i.e. the S-R relationship if the previous stimulus is always s

2. We select an arbitrary starting input probability assignment p^0

3. We compute the n-th S-R relationship as $P^n(y|x) = \sum_{s \in \mathcal{S}} p^n(s) P_s(y|x)$

4. The input probability assignment that is optimal given the S-R relationship (found using e.g. the cutting plane or Jimbo-Kunisawa algorithm) is then the $(n+1)$-th input probability assignment p_{n+1}.

Steps 3. and 4. are repeated until a self-consistent solution is found.

By comparing the efficiency after the first iteration with the efficiency after 5 iterations for different levels of inhibition (Fig A), we see the necessity of the described algorithm. Simply calculating the efficiency by the Jimbo-Kunisawa algorithm (i.e., stopping after the first iteration) could lead us to the incorrect conclusion that higher levels of inhibition lead to substantially higher efficiency.

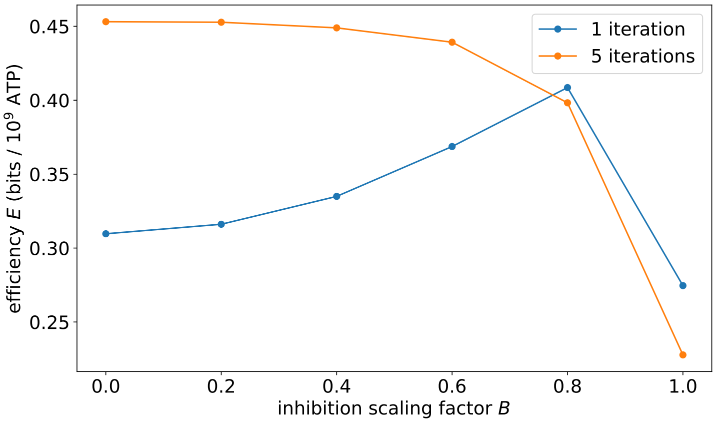

Figure A: Comparison of efficiencies calculated after 1 iteration of our algorithm and after 5 iterations. IB neuron (Table A in S1 Appendix), $\Delta = 100\,\mathrm{ms}$.

References

R. Blahut. Computation of channel capacity and rate-distortion functions. *IEEE Transactions on Information Theory*, 18(4):460–473, jul 1972. doi: 10.1109/tit.1972.1054855.

J. Huang and S. Meyn. Characterization and computation of optimal distributions for channel coding. *IEEE Transactions on Information Theory*, 51(7):2336–2351, jul 2005. doi: 10.1109/tit.2005.850108.

M. Jimbo and K. Kunisawa. An iteration method for calculating the relative capacity. *Information and Control*, 43(2):216–223, nov 1979. doi: 10.1016/s0019-9958(79)90719-8.

J. E. J. Kelley. The cutting-plane method for solving convex programs. *Journal of the Society for Industrial and Applied Mathematics*, 8(4):703–712, dec 1960. doi: 10.1137/0108053.

L. Kostal and P. Lansky. Information capacity and its approximations under metabolic cost in a simple homogeneous population of neurons. *Biosystems*, 112(3):265–275, jun 2013. doi: 10.1016/j.biosystems.2013.03.019.

P. Suksompong and T. Berger. Capacity analysis for integrate-and-fire neurons with descending action potential thresholds. *IEEE Transactions on Information Theory*, 56(2):838–851, feb 2010. doi: 10.1109/tit.2009.2037042.
