# Supplementary material for: The effect of inhibition on rate code efficiency indicators
Source: PLoS Comput Biol. 2019 Dec 2;15(12):e1007545. doi: 10.1371/journal.pcbi.1007545 (PMC6907877; doi:10.1371/journal.pcbi.1007545)
Supplement: S6 Appendix — (PDF) [file pcbi.1007545.s006.pdf]

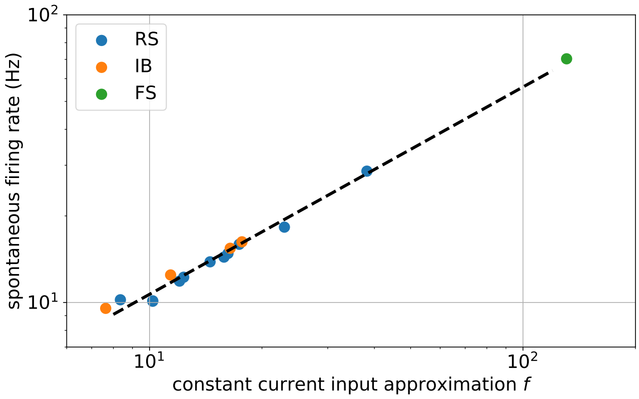


Figure A: The scatter plot compares the analytical calculation obtained from Eq (31). The dashed line represents a linear fit on the log-log plot providing the functional dependence between the prediction and the spontaneous firing rate.

# S6 Appendix.

**Approximation of spontaneous firing rate.**

Yamauchi et al. (2011) provide an approximate formula for the firing rate of the MAT model stimulated by a constant current $I$ (Eq (31)). In our case, the neurons are not stimulated by a constant current, but by a shot noise resulting in a fluctuating membrane potential. However, we found that by substituting $IR$ with $V_0 \doteq -63\,\mathrm{mV}$ this formula can be utilized to predict well the spontaneous firing rate for a large portion of the neurons (Fig A). The value $-63\,\mathrm{mV}$ roughly represents the usual value of the membrane potential when a spike is fired during the spontaneous activity. The formula in Eq (31) can provide predictions only for those parameter sets, for which $\omega > V_0$, thus excluding 15 neurons, generally with a negligible spontaneous activity. Moreover, the approximation does not apply for high firing rates, therefore also 3 FS neurons were excluded.

Linear fit in the Fig A provides us with a formula for the spontaneous firing rate $y_{\mathrm{spont}} \approx 10^{0.31} f^{0.72}$. Although the formula does not give a strict approximation of the spontaneous firing rate, it clearly explains almost all of the variance for the neurons with $\omega > V_0$, moreover, the dependence is monotonic. Therefore increase in $f$ will lead to an increase in the spontaneous firing rate $y_{\mathrm{spont}}$.

# References

S. Yamauchi, H. Kim, and S. Shinomoto. Elemental spiking neuron model for reproducing diverse firing patterns and predicting precise firing times. *Frontiers in Computational Neuroscience*, 5, 2011. doi: 10.3389/fncom.2011.00042.
