# Supplementary figures and images for: The effect of inhibition on rate code efficiency indicators
Source: PLoS Comput Biol. 2019 Dec 2;15(12):e1007545. doi: 10.1371/journal.pcbi.1007545 (PMC6907877; doi:10.1371/journal.pcbi.1007545)

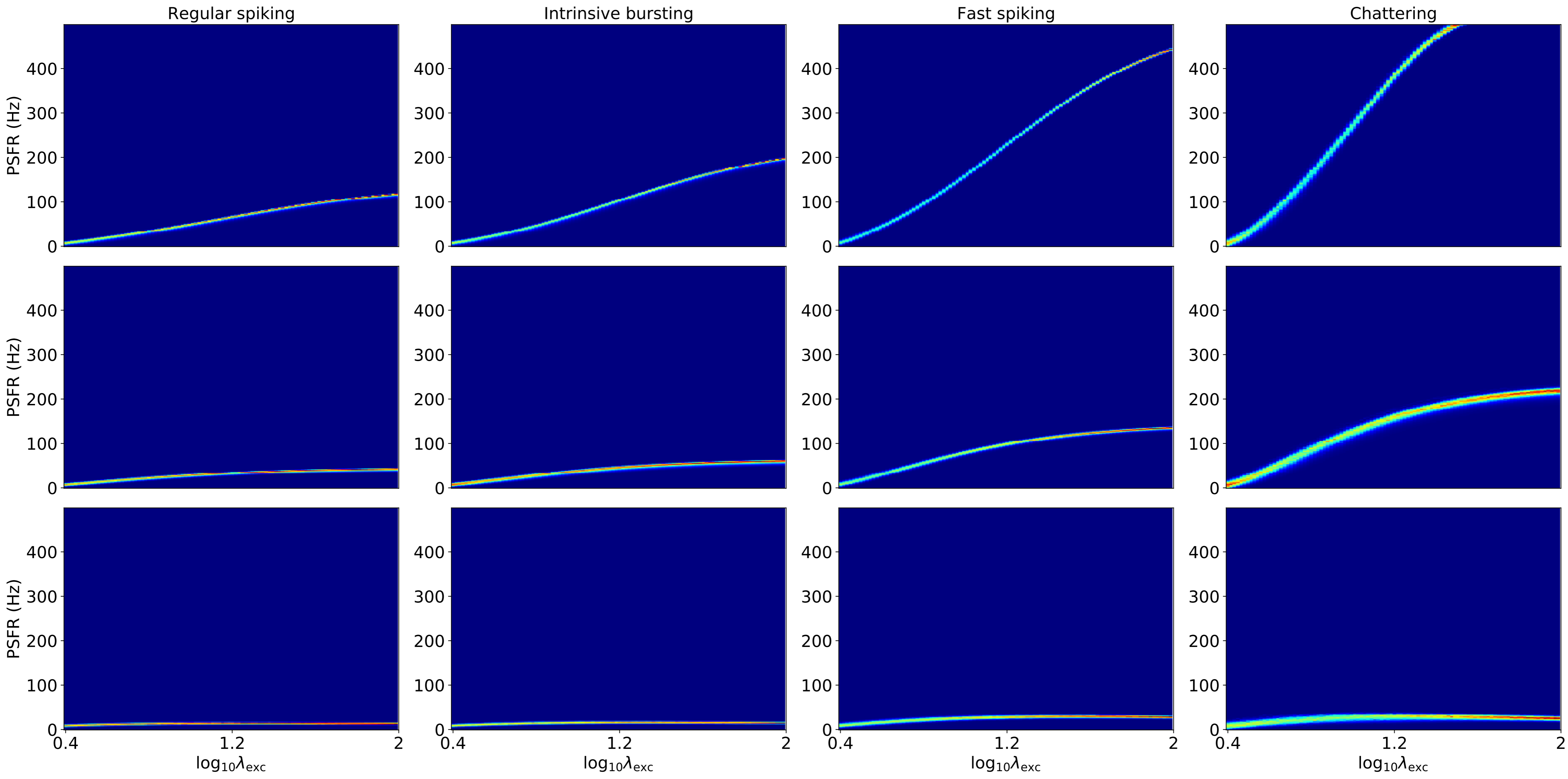

Supplement: S1 Fig — Same as Fig 2, but the scales are same for all the neurons and inhibition scaling factors B. Each row corresponds to a different inhibition regime. The ratio of inhibitory to excitatory conductance as a function of stimulus intensity is displayed in the leftmost column. The time window Δ was in this case chosen as 500 ms. The x-axis is logarithm of the rate of bombardment by excitatory synapses (Eq 23). The y-axis shows the post-synaptic firing rate (Eq 21). (PDF) [file pcbi.1007545.s007.pdf]
